# Background and Methodology of the 2024 annual survey from the panel ‘Health in Germany’ by the Robert Koch Institute

**DOI:** 10.25646/13572

**Published:** 2025-12-05

**Authors:** Johannes Lemcke, Stefan Damerow, Tim Kuttig, Ilter Öztürk, Stefan Albrecht, Tobias Heller, Sabine Born, Matthias Wetzstein, Jennifer Allen

**Affiliations:** Robert Koch Institute, Department of Epidemiology and Health Monitoring, Berlin, Germany

**Keywords:** Panel, Surveys and Questionnaires, Public Health, Adult, Mental Health, Health Behaviour, Health Literacy, Health Care, Social Determinants

## Abstract

**Background:**

As the RKI’s new data collection infrastructure, the RKI Panel ‘Health in Germany’ offers the opportunity to regularly collect primary data on topics relevant to public health among the population in Germany (survey mode: online – CAWI and written – PAPI). This article presents the participation rates for the sub-waves and the composition of the sample. It also describes the special features of the 2024 annual survey.

**Methods:**

The 2024 annual survey followed on from the initial recruitment study of the RKI panel. For this survey wave, the registered panel participants were surveyed in three sub-waves with four different questionnaires on different topics.

**Results:**

Participation rates between 81.3 % and 75.3 % were achieved in the three sub-waves of the 2024 annual survey. In the first sub-wave, 38,212 people participated, and in the second, 36,134 interviews were conducted. The third and final sub-wave comprised 35,786 interviews.

**Conclusions:**

The 2024 annual survey provides a comprehensive data basis for various public health-related issues. The following topics are covered: physical and mental health, use of healthcare services, health-related behaviour, health literacy, social conditions, and the relationship between climate change and health. The data refer to the German resident population aged 18 and over.

## 1. Introduction

As part of its health monitoring activities, the Robert Koch Institute (RKI) conducts extensive surveys and studies to collect epidemiological data on the health of the population in Germany. In the past, this primary data was collected in various study formats. Examples include earlier monitoring studies such as the German Health Interview and Examination Survey for Children and Adolescents (KiGGS) [1], the German Health Interview and Examination Survey for Adults (DEGS) [2], and the study German Health Update (GEDA) [3]. At the beginning of 2023, the RKI began the concrete development of a comprehensive panel infrastructure that can be used systematically and repeatedly in the future to collect survey, examination, and laboratory data. The establishment of the RKI Panel ‘Health in Germany’ thus responds to the high demand for current and regular primary data collection. Comparable infrastructures already exist in both German-speaking and international contexts, which have laid important groundwork. The panel is being developed in line with established panel infrastructures in the (social) sciences. National references include the Family Demographic Panel (FReDA), the Socio-Economic Panel (SOEP), and the GESIS Panel. Internationally, the Dutch LISS Panel and the Understanding America Study serve as important reference infrastructures. These examples (the panel infrastructures listed are only a selection) illustrate the practical and scientific benefits of mixed-mode panels that combine both online and postal surveys [4–12].

In the first stage of development, survey data will initially be collected in the RKI Panel ‘Health in Germany’. In further stages of development, examination and laboratory data will be integrated in the coming years. The main objective of the infrastructure being established is to generate reliable primary data on various topics relevant to health reporting (for a more detailed overview, including the planned stages of development, see [13]. This probabilistic panel infrastructure (i.e., based on a random sample of participants) was first implemented throughout Germany in 2024 and currently comprises around 47,000 registered panel participants aged 16 and older. These registered panel participants serve as the initial sample for the upcoming annual survey (for more details on the study design, see [13]). In the 2024 annual survey, key topics relating to the health status of the population were covered using four different questionnaires [14]. These included self-assessed physical health, the presence of chronic diseases, and physical functioning in everyday life. The surveys also focused on important mental and physical resources and symptoms, including health-related quality of life, general well-being, resilience, and common mental disorders such as depression and anxiety disorders. Another focus was on symptoms that have become more relevant in connection with the long-term effects of the COVID-19 pandemic, such as subjectively perceived memory and concentration problems, sleep disorders, and exhaustion. In addition, aspects of health care were collected, such as barriers to access and unmet care needs. Accidents and their personal and social consequences were also part of the survey. A new focus was placed on subjectively perceived stress caused by climate-related changes. In addition, in-depth surveys on health and nutrition literacy were conducted. Social factors such as social support and social isolation were also surveyed and can be considered in relation to physical and mental health. Sociodemographic characteristics such as educational and migration status, income, and working conditions were also recorded.

This article describes the methodology, study design, and specific aspects of the 2024 annual survey. It outlines the survey methods employed and the incentive schemes used. Response rates for the individual sub-waves are reported and stratified by relevant sociodemographic variables. In addition, the sample composition is presented differentiated by questionnaire and sub-wave. Key figures of the online survey process (such as completion time) are also provided. A more detailed description of the initial recruitment study can be found in the field report on the first recruitment study [15].

**Key messages**

► The 2024 annual survey from the RKI Panel ‘Health in Germany’ comprises three sub-waves.
► The participation rates for the sub-waves range between 81 % and 75 %.
► More than 47,000 registered panel participants were invited to take part in the 2024 annual survey.
► The composition of the panel sample remains stable across the sub-waves.
► The RKI panel uses online and paper questionnaires to accommodate different participation preferences.

## 2. Study design for the 2024 annual survey

### 2.1 Sample for the 2024 annual survey

#### Recruitment sample

Panel participants were recruited based on a random selection from population registers (of the respective residents’ registration offices; EMA for short) of 359 selected municipalities, using both the online survey mode (also known as Computer Assisted Web Interview, CAWI for short) and the written postal survey mode (also known as Paper and Pencil Interview, PAPI for short). A two-stage sampling design was implemented for recruitment. First, 359 primary sampling units (PSUs) were drawn from over 10,000 municipalities. The distribution of PSUs across the federal states was based on the population size of those over 16 years of age, with a minimum of 14 PSUs per federal state in order to adequately represent smaller states. Within the states, further stratification was carried out according to the BIK classification, a common standard for regional differentiation. Large cities were allocated multiple PSUs according to their population size.

In the second stage, the registration records of the EMA were used as the basis of random selection. For each PSU, 600 addresses were drawn, of which the RKI randomly selected 400; with the remaining 200 retained as reserves. The selection was stratified by age into eight age groups (16 – 85 and older) to ensure representative coverage of all age cohorts while accounting for expected participation rates. A more detailed description can be found in the field report on the first recruitment study [15].

A methodological peculiarity for the 2024 annual survey concerns the initial sampling for the panel recruitment study, in which a technical error occurred. In the age-stratified random sample from the addresses provided by the EMA, individual age cohorts were omitted in twelve municipalities. Consequently, some age groups were not represented in the recruitment sample. This mainly affected small municipalities, so the impact on the overall analysis within federal states is considered to be minor. An exception is the state of Berlin, where all districts are affected by the error, as there are no age cohorts aged 85 and older in the sample.

Following the completion of the initial recruitment, registered participants were randomly allocated to four approximately equal-sized groups and surveyed on a quarterly basis as part of a rotating study design (see section 2.2).

#### Sample for the 2024 annual survey

The gross sample (also called deployment sample) used in 2024 consisted of two participant groups distinguished by survey mode, which differ in terms of the procedural steps they underwent to be included in the panel:

► **CAWI panel participants:** After completing the recruitment study (the first survey before registration, also referred to as the ‘welcome survey’ for participants), CAWI participants who were willing to be surveyed again were redirected to the registration page of the panel portal. There, they provided contact details (name, email address, postal address) and demographic characteristics, including date of birth, sex at birth, and gender identity (see [16]), and created a user account. They then received an automatic verification email containing a confirmation link, completing their registration in the panel (double opt-in procedure). From this point on, online-registered participants were considered as active panel members, eligible for regular panel operations.
► **PAPI panel participants:** Participants registering for the panel via PAPI mode went through a technically modified registration process. Relevant contact information was imported into the panel management software (PAM) from the returned written declaration of consent to participate in the panel. At this stage, the offline participants in the panel were labeled as ‘offliners’.

Finally, a case verification was conducted for all registered panel participants, irrespective of survey mode. In this process, the information on year of birth, month of birth, and sex at birth was compared with the information from the population register. Cases showing substantial discrepancies between the two data sources were excluded. A more detailed description can be found in the methodology report on the first recruitment study [15].

A particular aspect of the 2024 annual survey concerns the subsequent inclusion of participants in the panel sample, also known as ‘latecomers’. Due to the tight schedule between the end of the recruitment phase and the start of the first annual survey, a portion of the originally planned initial panel sample was not yet fully validated at the time of first invitation. This affected around 21 % of the sample (approximately 10,000 individuals). In many of these cases, for example among PAPI participants, the written consent form for panel participation had not been validated in time. These individuals therefore received the invitation to participate in the first sub-wave in the second quarter at the scheduled time of the first reminder letter (the individuals received only one invitation letter and one reminder letter, i.e., no second reminder, as is usually the case). Further late inclusions resulted from errors in the case verification process (due, among other things, to incorrectly delivered data from the EMA regarding the month of birth), which checks whether the person who was contacted was actually the person who completed the questionnaire by comparing EMA data on the year of birth, birth month, and sex at birth with the corresponding self-reported data from the recruitment survey. Consequently, in the third sub-wave of the 2024 annual survey, an additional 1,101 individuals were added to the panel sample.

### 2.2 Wave design

**Figure 1:**
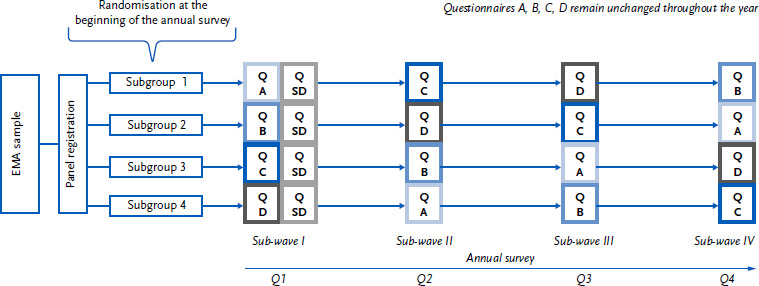
Schematic representation of the wave design of the 2024 annual survey of the RKI Panel ‘Health in Germany’ EMA = Residents’ Registration Office, Q = Questionnaire, SD = Sociode mographic Questionnaire

Within a regular annual survey, four sub-waves are conducted, each using one of four different questionnaires. The surveys are distributed evenly across four survey periods in order to control for seasonal effects on prevalence estimates. In order to rotate the four questionnaires, the sample was randomly divided into four equal subgroups after initial recruitment. Each subgroup then receives one of the four different questionnaires in rotating order at four different points in time. This design allows for the calculation of an annual estimate at the end of a survey period (in this case, one year). In addition to the four questionnaires, a separate sociodemographic questionnaire (Q-SD) was administered once in the first sub-wave. It was designed in both CAWI and PAPI modes so that it appeared as a coherent questionnaire together with the respective content of the main questionnaires (A, B, C, and D). Figure 1 illustrates the rotation of the four questionnaires A, B, C, and D, as well as the one-time collection of the Q-SD in the first sub-wave for all participants. The order of the questionnaires was deliberately arranged to reduce potential conditioning effects and thus minimise the influence of previous surveys on the responses. Due to delays in the recruitment process, only three of the planned four sub-waves could be realised for the 2024 annual survey.

Each questionnaire is designed to take no more than 20 minutes, resulting in a cumulative duration of up to 80 minutes across the four questionnaires per year. In the cover letters for each sub-wave, participants are informed that the survey will take approximately 15 to 20 minutes (per questionnaire). The Open Survey software was used for the online survey, and the PAM software from talk Online GmbH was used for the panel management.

#### Study field duration

The fieldwork periods for the sub-waves were as follows:

1st wave: May 28, 2024 to August 5, 2024
2nd wave: August 12, 2024 to October 14, 2024
3rd wave: October 28, 2024 to January 6, 2025

### 2.3 Sub-waves study design and incentives

#### Contact strategy

As outlined in the previous sections, panel participants can take part in either CAWI or PAPI survey mode. The respective survey mode was already assigned during the initial recruitment process and was based on the individual choice of the participants. Participants remain in the mode in which they registered for the panel. During the recruitment study, the sequence of survey modes offered was differentiated according to age groups:

► **Age groups 16 – 69 years:** Individuals in these age groups received a sequential mixed-mode design (push-to-web strategy). The invited individuals were initially given the option of participating in the survey online only. A paper questionnaire was only offered with the second reminder.
► **Age groups 70 years and older:** Individuals in these age groups received a simultaneous mixed-mode design, i.e., they were given the choice between CAWI and PAPI from the outset.

A change in the survey mode was not and is not currently planned.

CAWI and PAPI as survey modes offer important advantages over telephone and face-to-face surveys and therefore form the basis of the panel infrastructure. Online surveys are particularly characterised by their high speed and cost efficiency. Large numbers of participants can be reached in a short time without incurring printing or mailing costs (compared to surveys that send out multiple written reminders by post). In addition, technical functions such as filter questions or plausibility checks enable an improvement in data quality. Another advantage is flexibility, as participants can respond independently of time and place (within the field period) (see [17]). Written surveys are particularly suitable for people without internet access or with limited digital skills. They are often used in communication with older target groups. Official statistics show that around 96 % of people in Germany use the internet, but around 12 % of those aged between 65 and 74 are still offline [18]. Another advantage is that participants have more time to respond, which can lead to an increase in the quality of responses. In addition, written procedures are less susceptible to technical problems or data protection concerns (cf. [19]). The combination of both methods in so-called mixed-mode designs combines the aspects of speed and efficiency with better coverage of the age structure of the population. This allows the respective strengths to be optimally exploited (cf. [20]).

**Figure 2:**
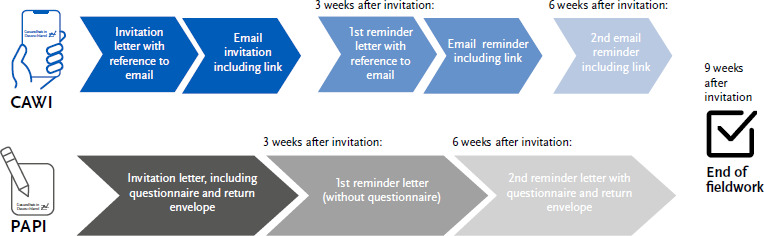
Study design sub-waves of the 2024 annual survey of the RKI Panel ‘Health in Germany’ CAWI = Computer Assisted Web Interview, PAPI = Paper and Pencil Interview

Each sub-wave followed the same study design, which is shown schematically in Figure 2. This study design differentiates according to survey mode. CAWI panel participants first received a written invitation letter announcing the sub-wave, which referred to a subsequent email invitation to the registered email address. The email invitation was sent approximately two days after the postal invitation letter was sent. After three weeks, a first reminder was sent, both in writing by post and by email. A second reminder was sent exclusively by email three weeks after the first reminder. The invitation process for PAPI panel participants was carried out exclusively by post (Figure 2). The infas Institut für angewandte Sozialwissenschaft GmbH was commissioned to handle postal dispatch of the participant documents and parts of the response management.

#### Incentives

Panel participants received an incentive (expense allowance) after or before participating in the sub-waves. The regular incentive scheme provided for CAWI participants to receive virtual incentive panel points (500 points, equivalent to € 5) after participating in a sub-wave (so-called post-paid incentives). These could then be exchanged by the CAWI panel participants themselves for vouchers of their choice in the panel incentive shop of the online panel portal. PAPI participants received € 5 in cash in a thank-you letter after participating in a sub-wave.

**Figure 3:**
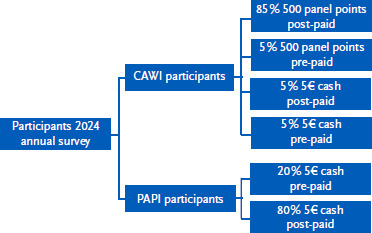
Incentive experiment in the 2024 annual survey of the RKI Panel ‘Health in Germany’

As part of an incentive experiment, which was carried out once for the entire annual survey (i.e., for each sub-wave), 5 % of CAWI panel participants received the incentive panel points before participating in the sub-wave, i.e., with the invitation (so-called pre-paid incentives). This was explicitly pointed out to them in the invitation. A further 10 % of CAWI-panel participants received a € 5 cash incentive by means of a thank-you letter sent by post, with one half receiving it in advance and the other half after participating in the sub-wave. These experimental groups were assigned randomly. An experiment was also integrated for PAPI participants for the entire annual survey. 20 % of PAPI panel participants received a € 5 cash incentive in advance together with the invitation for the respective sub-wave. The remaining 80 % of PAPI panel participants received the € 5 cash incentive conditionally only after successfully participating in the sub-wave in a thank-you letter sent by post. Figure 3 shows the structure of the incentive experiment. A separate publication will be released with further background information, the objectives, and the results of this incentive experiment.

### 2.4 Calculation of participation rates

This article reports response rates and participation rates in accordance with the standards of the American Association for Public Opinion Research (AAPOR) [21]. The following figures show the participation rates broken down by sub-wave and questionnaire. The baseline varies in each case. First, the participation rates are shown in relation to the registered panel participants. According to the AAPOR classification, this corresponds to the response rate RR2. The AAPOR participation rate (RR2) is the number of respondents who have completed the sub-wave questionnaire in full or in part, divided by the number of all panel participants who received an invitation to participate in the sub-wave. Within the panel context, questionnaires are considered partially completed if at least the declaration of consent was provided and at least one content item was answered.

An important difference from the participation rates during the initial recruitment study is that, by definition, there are no cases with unknown eligibility status in the panel context as defined by the AAPOR category ‘UE – Unknown Eligibility’. The reason for this is that, starting with the first sub-wave, only participants for whom valid postal and email addresses were already known from the recruitment process are contacted. For this reason, the AAPOR category ‘UE’ only appears in the recruitment study (not shown in this article). In the following, the AAPOR RR2 participation rate is also stratified by age, sex, education, and survey mode. For better comparability between CAWI and PAPI, the Annex Table 1, Annex Table 2, and Annex Table 3 also show the AAPOR RR1 participation rate for the three sub-waves. This uses the same case definition for both modes (only fully completed questionnaires), as there are technically no partially completed questionnaires in PAPI mode.

The second basis for the reported participation rates is the recruitment study. Here, the cumulative response rate (CUMRR) is reported. The CUMRR2 (which includes both completed and partially completed questionnaires), as defined by AAPOR, is calculated as the product of a panel’s recruitment rate and the participation rate for the current survey wave. With this second basis, the potential non-response bias can be better estimated in relation to recruitment, even if the participation rates themselves are not an exhaustive indicator for quantifying non-response bias [22]. For this reason, the sample composition is explained in the next section as a further quality indicator.

When presenting participation rates in the panel context, it should be noted that the gross sample (i.e., the initial sample of all panel participants active in the respective sub-wave) is reduced due to deletions, revocations, and deaths. In addition, the special feature described above of subsequent inclusions in the respective sub-wave increased the gross sample size.

### 2.5 Presentation of the sample composition

To assess the sample composition, the distribution is differentiated according to age, sex, federal state, education (Comparative Analysis of Social Mobility in Industrial Nations, CASMIN [23]) and household size (single-person vs. multi-person household) and presented according to sub-wave and questionnaire and compared with reference distributions from population statistics [24] and the 2021 microcensus [25]. When differentiating by questionnaire, the proportions are additionally calculated taking into account the sample weights. It should be noted that the weights are only defined for persons aged 18 and over. For this reason, the table only shows persons aged 18 and over. The analyses were performed using R (version 4.3.0), and the two-stage sample design was taken into account when calculating the weighted proportions using the survey package [26]. It should be noted that a sample increase was integrated into the recruitment study in the states of Berlin and Schleswig-Holstein. The sample augmentations were carried out at the request of the states and were financed by them.

### 2.6 Weighting

In order to correct distortions due to selective participation and deviations of the sample from the population structure as far as possible, a multi-stage sample weight was calculated. It first takes into account the sample weight of the initial recruitment study. In a dropout weighting, dropout weights were calculated using the data from the recruitment study to counteract selective participation in the repeated sub-waves. Finally, adjustments were calculated to reflect the population update as of December 31, 2023, and the 2021 microcensus. This takes into account age, sex, federal state, BIK municipality type [27], educational groups according to CASMIN [23] and household size (single vs. multi-person household). The weighting was calculated separately for each questionnaire variant and is only defined for ages 18 and above. A detailed methodological description will follow in a separate article [28].

## 3. Results

Before presenting the results, it should be noted that the composition of the samples is only reported for individuals aged 17 and above. Although 16-year-olds were also included in the recruitment process, the proportion of 16-year-olds is too small to allow for reliable content analysis due to the aging of the sample between recruitment and the time of the survey.

### 3.1 Key figures for the survey process

For CAWI participants, the use of paradata (i.e., data generated during the survey process) allows for a more accurate analysis of completion behaviour. The average completion time varies significantly between questionnaires. When calculating the completion times, individual questions with a completion time of more than ten minutes were not taken into account. Participants took the longest to complete questionnaire C (average: 20.9 minutes; standard deviation: 10.7 minutes; median: 18.4 minutes; 10th percentile: 11.0 minutes; 90th percentile: 33.2 minutes), while they were faster in questionnaires A (average: 8.1 minutes; standard deviation: 4.4 minutes; median: 7.0 minutes; 10th percentile: 4.0 minutes; 90th percentile: 13.5 minutes), B (average: 13.7 minutes; standard deviation: 6.6 minutes; median: 12.1 minutes; 10th percentile: 7.5 minutes; 90th percentile: 21.6 minutes) and D (average: 7.2 minutes; standard deviation: 4.4 minutes; median: 6.0 minutes; 10th percentile: 3.3 minutes; 90th percentile: 12.7 minutes). The main reasons for this are the topics covered in the questionnaires and the associated filtering. Questionnaire C dealt with the topic of mental health, with almost all items being presented in full to all participants. The other questionnaires contained topic-specific blocks of questions that were only presented to a comparatively small proportion of the panel participants, depending on their answers to preceding filter questions (e.g., on the topics of diabetes or accidental injuries). The sociodemographic questionnaire surveyed in the first sub-wave took relatively little time to complete (average: 3.4 minutes; standard deviation: 2.3 minutes; median: 2.8 minutes; 10th percentile: 1.6 minutes; 90th percentile: 5.7 minutes), so that the target maximum survey duration of approximately 20 minutes could be adhered to in most cases.

Most CAWI participants used a smartphone to participate (58.6 %), while slightly fewer completed the questionnaires on a desktop PC or notebook (40.7 %). The use of tablets was very low in comparison (0.7 %).

### 3.2 Participation rates

In sub-wave 1, 46,977 panel participants were invited to take part in the survey, 46,851 in sub-wave 2, and 47,535 in sub-wave 3. The reduction in the gross sample from wave 1 to wave 2 is due to deletion requests and revocations of consent to be re-surveyed by registered panel participants. The increase in the gross sample in the third sub-wave is due to the addition of so-called latecomers to the panel (this is explained in more detail in section 2.6).

The figures in this report are based on the gross sample of the respective sub-waves. Due to subsequent deletions, revocations of consent (both at the request of the participants) and further data curation processes, the figures may differ slightly from the current version of the evaluable data sets.

Table 1 shows the participation rates according to RR2. Following the AAPOR system, this means that partially completed interviews are also considered in this calculation. The recruitment rate of 28.7 % achieved in the recruitment sample was used as the basis for calculating the CUMRR2. A comparison of the questionnaires between the sub-waves reveals no consistent pattern in the participation rates. However, it is striking that questionnaire A has the second-highest participation rate in sub-wave 1 at 81.8 %, but the lowest in sub-wave 3 at 73.3 %. Questionnaire D shows the highest participation rate of 81.9 % in sub-wave 1, but falls to 75.7 % in sub-wave 3. The overall participation rate for RR2 is 81.3 % in sub-wave 1 and drops to 75.3 % in sub-wave 3. Overall, a decline in participation rates across waves can be observed for all questionnaires – a typical pattern in panel surveys. The cumulative response rate (CUMRR 2) reflects this pattern, except that this rate was multiplied by the initial recruitment rate of the recruitment study.

Table 2 shows the participation rate RR2 broken down by age, sex, survey mode, and education. There has been a decline in the overall participation rate across the various waves (row ‘Interviews’ in Table 2).

When stratified by age, it becomes clear that older age groups tend to have higher participation rates. The 60 to 69 age group had the highest participation rate in the first sub-wave at 89.5 % and remained stable across the sub-waves. In contrast, the younger age groups show the lowest participation rates, especially the 17- to 29-year-olds with 71.5 % in sub-wave 1. The decline in participation rates is also most evident in this age group. In the third sub-wave, the participation rate is still 59.3 %. The analysis differentiated by sex shows that women consistently have higher participation rates. In the first sub-wave, the participation rate for women is 83.3 %, while the participation rate for men is 79.1 %. However, both sexes show a decline in participation rates across the subsequent sub-waves.

Looking at the survey methods, the CAWI mode shows a participation rate of 81.4 % in sub-wave 1, while the PAPI mode is 81.3 % in sub-wave 1. The CAWI mode shows a decline in the following waves. In contrast, the participation rate of PAPI participants remains constant or increases slightly across the sub-waves. Finally, the analysis by educational level shows that people with a high level of education have the highest participation rates, at 84.3 % in sub-wave 1. In contrast, people with a low level of education have the lowest participation rates, at 79.2 % in the first sub-wave. Here, too, a decline in participation rates can be observed across the waves. The decline per sub-wave between the first and second sub-waves is between four and five percentage points in all three education groups, at a similar level. After that, participation rates appear to stabilise, with the decline in percentage points from the second to the third sub-wave being one to three percentage points lower.

### 3.3 Sample composition

Table 3 shows the sample composition of the total number of registered participants in 2024 and the respective samples of participants in sub-waves 1 to 3. A comparison between the reference distributions and the registered participants shows deviations in all parameters examined, with the largest differences being found in German citizenship, educational distribution, and individual federal states. There are no substantial differences in the distributions between the samples of the individual sub-waves and the total number of registered participants. This means that the composition of the panel remains largely stable after registration and during the sub-waves. However, there is a greater difference in the age groups. In the youngest age group of 17- to 29-year-olds, the proportion within the panel declines most sharply over time.

Table 4 shows the unweighted and weighted distributions of participants in questionnaires A to D. There are no differences between the individual questionnaires, and the results do not differ from those differentiated by sub-waves. Taking into account the sample weighting, there are no substantial differences from the reference distributions, with the exception of German nationality. The differences decrease only slightly by about one percentage point, but the deviation is still substantially high at around nine percentage points.

## 4. Discussion

### 4.1 Participation rates

Based on the participation rates, the first annual survey from the ‘Health in Germany’ panel (RKI Panel 2024) indicates a robust willingness among panel participants to participate again. The participation rates of between 81 % and 75 % based on the registered panel participants invited in each wave are above the previously anticipated range (in the published power estimate, which was done in the course of the panel design, a conservative average participation rate of around 65 % was assumed; see [13]). Compared with participation rates for similar panel infrastructures, these values are in the middle range [4]. The decline in the total number of panel participants, i.e., those who did not participate in a single wave (known as passive panel attrition), is also to be expected and normal for panel studies [29]. Around 12 % of the invited registered panel participants did not participate in any wave of the 2024 annual survey. A differentiated analysis of participation rates depending on the questionnaire used indicates possible sequence effects. For example, questionnaire A has the second-highest participation rate in the first sub-wave and the lowest in the third sub-wave. This could be due to the sequence of the questionnaires. It is conceivable that specific thematic content influences willingness to participate in subsequent sub-waves. In addition, the length or perceived time required to complete individual questionnaires could also play a role in the willingness to participate. Whether certain topics or the scope of individual questionnaires actually have an impact on participation in subsequent surveys will be the subject of future analyses. The results could provide valuable insights for the content design and sequencing of survey instruments in panel studies in the future.

As expected, participation rates declined over the course of the three waves. In this context the RKI Panel ‘Health in Germany’ also shows that it is difficult to retain younger age groups (under 30) in the panel. There is also a consistently higher participation rate among women than men (at this descriptive level). These findings largely coincide with other epidemiological studies, which have shown that the probability of participation correlates significantly with sociodemo-graphic factors. These studies also show that older people (up to a certain age), women, and people with a higher socio-economic status (higher income and higher education) are more likely to participate [30–33]. The decline in the younger age group can be easily explained, primarily by age-specific living environments, such as more frequent changes of residence [34] or the disproportionately high proportion of single households (which can sometimes make contact more difficult). However, these possible explanations must also be followed by age-specific panel maintenance measures (such as the form of incentives or other contact sequences). One advantage of the RKI panel in this context could be that a fairly high CAWI share of around 95 % can be observed in this age group. This could accommodate the potentially higher need for flexible and individual survey modes in this age group. The downside of this more flexible form of participation can also be a higher degree of non-commitment. However, these interpretations must initially be viewed with caution, as confounding effects – such as a mode effect in connection with the incentives offered – could also influence the probability of participation. This interpretation seems plausible when looking at the different participation rates by survey mode. For PAPI participants, the participation rates are at an almost constant level. Due to the design, the majority of PAPI participants are 70 years of age or older. Age per se seems to have a significant influence on willingness to participate. However, the group of PAPI participants was also incentivised with cash. Our own studies and a large number of methodological studies have shown that this has a significant influence on the probability of participation[35, 36]. In future refresher samples, the different probabilities of participation can be taken into account by oversampling younger age groups (e.g., 16- to 29-year-olds). However, it should be noted that the associated higher design weight of this group may reduce the effective sample size overall.

### 4.2 Sample composition

A comparison of the sample composition with official data reveals substantial deviations, particularly with regard to education and nationality. The differences are comparable to or smaller than those found in previous RKI studies (compared to GEDA 2019/2020-EHIS, there is a smaller education bias) [3]. In this context, educational bias refers to a potentially distorted sample due to a higher or lower probability of participation by certain educational groups (in this case, a significantly lower probability of participation by people with low levels of education and a significantly higher probability of participation by people with high levels of education). When comparing the RKI panel and the sample used in the 2024 annual survey with other large population-based samples, it is noticeable that the educational bias is within an average and now expected range [33]. Differences in the proportions of the federal states are not due to selective participation effects, but are design-related. In the sampling process, samples in small states were topped up to ensure a minimum number of cases for the state-specific analyses. In addition, there were extra sample increases for the states of Berlin and Schleswig-Holstein. It appears that the sample weighting can largely compensate for the existing deviations. One exception is German citizenship, which highlights a substantial underrepresentation of persons of non-German citizenship. In principle, population figures on German citizenship are available and could be taken into account in the sample weighting. However, the study design does not currently provide for methods such as multilingual questionnaires that would enable non-German-speaking persons to participate in the study. This means that certain and potentially relevant population groups within the subgroup of persons without German citizenship are excluded. Since this exclusion is not random, adjusting the sample with regard to German citizenship would potentially exacerbate existing biases within this group. For future refresher samples, targeted measures are planned to improve the inclusion of non-German-speaking population groups. This includes, in particular, the provision of questionnaires in additional languages. This measure can improve the sample composition with regard to nationality and thus increase the representativeness of the sample. In the RKI study GEDA Fokus, the provision of multilingual questionnaires made it possible to better integrate the group of people with a short length of stay into the sample [37].

### 4.3 Special features of the 2024 annual survey

The special features of the 2024 annual survey have already been explained in the section on study design. The resulting limitations are described in more detail below. The omission of a sub-wave for the first quarter means that the potential seasonal effects mentioned above cannot be taken into account, at least for this quarter. In addition, this reduces the effective number of cases available per questionnaire, which may have a direct impact on the statistical power of the analyses. However, the rotating study design allowed each of the four questionnaires to be used in the remaining three sub-waves, which meant that relevant data could still be generated. This resulted in a sample size of approximately n = 27,000 for each questionnaire, despite the omission of the first sub-wave. Due to the higher participation rates in the initial recruitment and participation in the sub-waves, this case number exceeds the originally anticipated case numbers of approximately 14,500 participants per sub-wave [13].

The error in the sampling of the EMA sample in the different age cohorts means that, especially for Berlin (a federal state that is also integrated with a supplementary sample), evaluations in the 2024 annual survey with persons aged 80 and over are not possible. As a consequence, a further recruitment study was successfully carried out for the municipalities concerned, in particular for Berlin, with the aim of closing the gaps in the age cohorts. These newly recruited panel participants were integrated into the panel infrastructure and are thus a regular part of the 2025 annual survey.

In summary, it can be said that the first annual survey of the RKI Panel ‘Health in Germany’ provides a comprehensive data basis on selected important topics relating to physical and mental health, health behaviour, health risks, and health care for the population aged 18 and over. Future annual surveys will expand this database to include additional topics, and the panel approach will enable longitudinal analyses for some topics. This will create an even more comprehensive data source that provides reliable data on a wide range of topics for evidence-based public health.

## Figures and Tables

**Table 1:** Participation rates Response Rate 2 (RR2) by sub-wave and questionnaire. Source: 2024 annual survey from the RKI Panel ‘Health in Germany’

Questionnaire	Sub-wave 1			Sub-wave 2			Sub-wave 3			Total		
	n	RR2 (%)	CUMRR2 (%)	n	RR2 (%)	CUMRR2 (%)	n	RR2 (%)	CUMRR2 (%)	n	RR2 (%)	CUMRR2 (%)
A	9,567	81.8	23.5	9,289	79.0	22.7	8,690	73.3	21.0	27,546	78.0	22.4
B	9,550	81.2	23.3	8,772	75.1	21.6	9,161	76.8	22.0	27,483	77.7	22.3
C	9,444	80.5	23.1	9,050	77.5	22.2	8,974	75.4	21.6	27,468	77.8	22.3
D	9,651	81.9	23.5	9,023	76.9	22.1	8,961	75.7	21.7	27,635	78.1	22.4
Total	38,212	81.3	23.3	36,134	77.1	22.1	35,786	75.3	21.6			

Note: Response Rate 2 (RR2) was calculated according to the AAPOR Standard Definitions [21]

RR2 = Response Rate 2, CUMRR2 = cumulative response rate 2, AAPOR = American Association for Public Opinion Research

**Table 2:** Response Rate 2 (RR2) by sub-wave and by age, sex, survey mode, and education. Source: 2024 annual survey from the RKI Panel ‘Health in Germany panel’

Characteristics	Sub-wave 1		Sub-wave 2		Sub-wave 3	
	n	RR2 (%)	n	RR2 (%)	n	RR2 (%)
Interviews	38,212	81.3	36,134	77.1	35,786	75.3
**Age**						
17 – 29 years	6,088	71.5	5,291	62.3	5,129	59.3
30 – 39 years	5,540	75.4	5,094	69.4	5,035	67.3
40 – 49 years	4,570	78.7	4,282	73.8	4,281	72.1
50 – 59 years	6,895	84.5	6,621	81.2	6,618	79.6
60 – 69 years	6,842	89.5	6,665	87.3	6,654	85.7
70 – 79 years	5,132	89.3	5,056	88.5	5,031	87.9
≥ 80 years	3,145	83.7	3,125	84.2	3,038	82.8
**Sex**						
Female	20,879	83.3	19,782	79.1	19,532	77.0
Male	17,333	79.1	16,352	74.9	16,254	73.3
**Survey mode**						
CAWI	29,418	81.4	27,267	75.5	27,121	73.6
PAPI	8,794	81.3	8,867	82.5	8,665	81.2
**Education (CASMIN)**						
Low	7,424	79.2	7,075	75.5	6,918	73.0
Medium	18,307	81.1	17,245	76.5	17,019	74.0
High	12,251	84.3	11,689	80.5	11,764	79.5

Note: The slightly lower number of cases in the presentation of the education groups is due to missing information in the survey of educational attainment.

RR2 = Response Rate 2, CASMIN = Comparative Analysis of Social Mobility in Industrial Nations

**Table 3:** Sample composition of selected parameters compared to reference distributions, differentiated by panel 2024 total and sub-waves 1 to 3. Source: 2024 annual survey from the RKI Panel ‘Health in Germany’

Parameter	Reference (%)	Panel (%) (Δ)^a^	Sub-wave 1 (%) (Δ)^a^	Sub-wave 2 (%) (Δ)^a^	Sub-wave 3 (%) (Δ)^a^	Panel n	Sub-wave 1 n	Sub-wave 2 n	Sub-wave 3 n
**Sex** ^b^									
Female	51.0	53.3 (2.3)	54.6 (3.6)	54.8 (3.7)	54.6 (3.5)	25,521	20,879	19,782	19,532
Male	49.0	46.7 (- 2.3)	45.4 (- 3.6)	45.2 (- 3.7)	45.4 (- 3.5)	22,342	17,333	16,352	16,254
**Age** ^b^									
17 – 29 years	16.9	18.1 (1.2)	15.9 (- 1.0)	14.6 (- 2.2)	14.3 (- 2.5)	8,677	6,088	5,291	5,129
30 – 39 years	15.7	15.7 (0.0)	14.5 (- 1.2)	14.1 (- 1.6)	14.1 (- 1.6)	7,509	5,540	5,094	5,035
40 – 49 years	14.5	12.4 (- 2.1)	12.0 (- 2.6)	11.8 (- 2.7)	12.0 (- 2.6)	5,955	4,570	4,282	4,281
50 – 59 years	17.4	17.4 (0.1)	18.0 (0.7)	18.3 (0.9)	18.5 (1.1)	8,346	6,895	6,621	6,618
60 – 69 years	16.3	16.3 (0.0)	17.9 (1.6)	18.4 (2.2)	18.6 (2.3)	7,797	6,842	6,665	6,654
70 – 79 years	10.6	12.1 (1.5)	13.4 (2.8)	14.0 (3.4)	14.1 (3.5)	5,800	5,132	5,056	5,031
≥ 80 years	8.6	7.9 (- 0.7)	8.2 (- 0.4)	8.6 (0.0)	8.5 (- 0.2)	3,779	3,145	3,125	3,038
**Education (CASMIN)** ^c^									
Low	34.4	20.1 (- 14.3)	19.6 (- 14.8)	19.7 (- 14.7)	19.4 (- 15.0)	9,510	7,424	7,075	6,918
Medium	45.3	48.6 (3.3)	48.2 (2.9)	47.9 (2.6)	47.7 (2.4)	23,027	18,307	17,245	17,019
High	20.3	31.3 (11.0)	32.2 (11.9)	32.5 (12.1)	33.0 (12.6)	14,816	12,251	11,689	11,764
**Federal state** ^b^									
Baden-Württemberg	13.3	9.8 (- 3.6)	9.5 (- 3.9)	9.5 (- 3.8)	10.0 (- 3.3)	4,613	3,592	3,418	3,577
Bavaria	15.9	11.9 (- 3.9)	12.4 (- 3.5)	12.3 (- 3.5)	12.2 (- 3.7)	5,637	4,708	4,431	4,350
Berlin^d^	4.5	10.2 (5.7)	10.1 (5.6)	9.8 (5.3)	9.7 (5.2)	4,808	3,839	3,515	3,461
Brandenburg	3.1	3.2 (0.2)	3.2 (0.2)	3.3 (0.2)	3.2 (0.2)	1,540	1,225	1,177	1,153
Bremen	0.8	3.2 (2.4)	3.2 (2.4)	3.2 (2.4)	3.1 (2.3)	1,510	1,209	1,143	1,120
Hamburg	2.2	3.7 (1.4)	3.6 (1.4)	3.6 (1.4)	3.6 (1.3)	1,743	1,386	1,315	1,271
Hesse	7.6	5.3 (- 2.2)	4.7 (- 2.9)	4.8 (- 2.8)	5.3 (- 2.3)	2,516	1,768	1,709	1,894
Mecklenburg-Western Pomerania	2.0	3.1 (1.1)	3.1 (1.1)	3.0 (1.1)	3.0 (1.0)	1,449	1,176	1,095	1,070
Lower Saxony	9.6	7.5 (- 2.1)	7.4 (- 2.2)	7.5 (- 2.1)	7.6 (- 2.1)	3,534	2,823	2,713	2,702
North Rhine-Westphalia	21.4	15.8 (- 5.6)	15.8 (- 5.6)	15.8 (- 5.6)	15.7 (- 5.7)	7,473	6,007	5,697	5,607
Rhineland-Palatinate	4.9	3.6 (- 1.3)	3.8 (- 1.2)	3.8 (- 1.1)	3.7 (- 1.2)	1,726	1,424	1,364	1,331
Saarland	1.2	3.2 (2.0)	3.3 (2.1)	3.3 (2.1)	3.2 (2.0)	1,529	1,249	1,188	1,149
Saxony	4.9	3.7 (- 1.2)	3.8 (- 1.1)	3.8 (- 1.0)	3.8 (- 1.1)	1,733	1,431	1,383	1,348
Saxony-Anhalt	2.6	3.1 (0.5)	3.1 (0.5)	3.2 (0.5)	3.1 (0.5)	1,468	1,193	1,138	1,104
Schleswig-Holstein^d^	3.5	9.8 (6.3)	9.9 (6.4)	10.0 (6.5)	9.8 (6.3)	4,627	3,767	3,594	3,503
Thuringia	2.5	3.0 (0.5)	3.0 (0.5)	3.1 (0.6)	3.0 (0.5)	1,414	1,158	1,129	1,080
**German citizenship** ^b^									
Yes	85.0	94.6 (9.6)	95.6 (10.6)	96.0 (11.1)	96.1 (11.2)	44,713	36,254	34,552	34,311
No	15.0	5.4 (- 9.6)	4.4 (- 10.6)	4.0 (- 11.1)	3.9 (- 11.2)	2,561	1,663	1,422	1,377
**Household size** ^c^									
Single-person household	25.4	21.2 (- 4.2)	21.3 (- 4.1)	21.2 (- 4.2)	21.1 (- 4.3)	10,008	8,064	7,633	7,539
Multi-person household	74.6	78.8 (4.2)	78.7 (4.1)	78.8 (4.2)	78.9 (4.3)	37,215	29,824	28,312	28,120

^a^Deviations from the reference value in percentage points

^b^Reference values according to population statistics for 2022

^c^Reference values according to the 2021 microcensus

^d^Additional samples were integrated for the federal states of Berlin and Schleswig-Holstein

CASMIN = Comparative Analysis of Social Mobility in Industrial Nations

**Table 4:** Sample composition of selected parameters compared to reference distributions differentiated by questionnaire types A to D. unweighted and weighted aged 18 and over. Source: 2024 annual survey from the RKI Panel ‘Health in Germany’

Parameter	Reference	Q A (%) (Δ)^a^	Q B (%) (Δ)^a^	Q C (%) (Δ)^a^	Q D (%) (Δ)^a^	Q A weighted (%) (Δ)^a^	Q B weighted (%) (Δ)^a^	Q C weighted (%) (Δ)^a^	Q D weighted (%) (Δ)^a^	Q A N	Q B N	Q C N	Q D N
**Sex^b^**													
Female	51.0	54.5 (3.5)	54.8 (3.8)	54.5 (3.4)	54.7 (3.6)	51.1 (0.0)	51.1 (0.0)	51.1 (0.1)	51.1 (0.1)	14,822	14,881	14,762	14,926
Male	49.0	45.5 (- 3.4)	45.2 (- 3.8)	45.5 (- 3.4)	45.3 (- 3.6)	48.9 (0.0)	48.9 (0.0)	48.9 (- 0.1)	48.9 (- 0.1)	12,377	12,266	12,340	12,380
**Age^b^**													
18 – 29 years	16.9	14.2 (- 2.6)	14.0 (- 2.9)	14.1 (- 2.7)	14.5 (- 2.4)	16.0 (- 0.9)	15.9 (- 1.0)	15.9 (- 1.0)	15.9 (- 1.0)	3,870	3,803	3,829	3,954
30 – 39 years	15.7	14.2 (- 1.4)	14.5 (- 1.2)	14.5 (- 1.2)	14.4 (- 1.3)	15.8 (0.2)	15.9 (0.2)	15.9 (0.2)	15.9 (0.2)	3,874	3,927	3,933	3,926
40 – 49 years	14.5	12.1 (- 2.4)	12.0 (- 2.5)	12.1 (- 2.5)	12.1 (- 2.5)	14.7 (0.2)	14.7 (0.2)	14.7 (0.2)	14.7 (0.2)	3,290	3,263	3,268	3,294
50 – 59 years	17.4	18.6 (1.3)	18.6 (1.2)	18.4 (1.0)	18.3 (0.9)	17.6 (0.2)	17.6 (0.2)	17.6 (0.2)	17.6 (0.2)	5,072	5,046	4,989	5,000
60 – 69 years	16.3	18.4 (2.1)	18.6 (2.3)	18.6 (2.3)	18.5 (2.2)	16.5 (0.2)	16.5 (0.2)	16.5 (0.2)	16.5 (0.2)	5,007	5,035	5,032	5,048
70 – 79 years	10.6	14.0 (3.4)	14.0 (3.4)	13.9 (3.3)	13.8 (3.1)	10.7 (0.1)	10.7 (0.1)	10.7 (0.1)	10.7 (0.1)	3,811	3,793	3,765	3,758
≥ 80 years	8.6	8.4 (- 0.3)	8.4 (- 0.2)	8.4 (- 0.2)	8.5 (- 0.1)	8.7 (0.1)	8.7 (0.1)	8.7 (0.1)	8.7 (0.1)	2,275	2,280	2,286	2,326
**Education (CASMIN)^c^**													
Low	34.4	19.2 (- 15.1)	18.9 (- 15.4)	19.2 (- 15.1)	19.2 (- 15.1)	33.4 (- 1.0)	33.2 (- 1.1)	33.3 (- 1.1)	33.2 (- 1.2)	5,227	5,132	5,200	5,236
Medium	45.3	47.8 (2.5)	48.4 (3.1)	47.9 (2.6)	47.9 (2.6)	46.4 (1.1)	46.5 (1.1)	46.4 (1.1)	46.5 (1.2)	12,978	13,107	12,964	13,043
High	20.3	32.9 (12.6)	32.7 (12.4)	32.9 (12.5)	32.9 (12.6)	20.3 (-0.1)	20.3 (0.0)	20.3 (0.0)	20.3 (0.0)	8,944	8,861	8,889	8,973
**Bundesland** ^b^													
Baden-Württemberg	13.3	9.6 (- 3.7)	9.7 (- 3.7)	9.7 (- 3.7)	9.6 (- 3.7)	13.3 (0.0)	13.3 (0.0)	13.4 (0.0)	13.4 (0.0)	2,615	2,617	2,614	2,622
Bavaria	15.9	12.3 (- 3.6)	12.4 (- 3.5)	12.1 (- 3.7)	12.3 (- 3.5)	15.9 (0.0)	15.9 (0.0)	15.9 (0.0)	15.9 (0.0)	3,339	3,352	3,278	3,356
Berlin^d^	4.5	9.8 (5.3)	9.8 (5.3)	9.8 (5.4)	9.9 (5.4)	4.5 (0.0)	4.5 (0.0)	4.5 (0.0)	4.5 (0.0)	2,667	2,658	2,658	2,688
Brandenburg	3.1	3.2 (0.1)	3.2 (0.1)	3.3 (0.3)	3.3 (0.2)	3.1 (0.0)	3.1 (0.0)	3.1 (0.0)	3.1 (0.0)	872	858	900	893
Bremen	0.8	3.2 (2.4)	3.2 (2.4)	3.2 (2.4)	3.1 (2.3)	0.8 (0.0)	0.8 (0.0)	0.8 (0.0)	0.8 (0.0)	862	867	864	849
Hamburg	2.2	3.6 (1.3)	3.6 (1.3)	3.6 (1.4)	3.7 (1.5)	2.2 (0.0)	2.2 (0.0)	2.2 (0.0)	2.2 (0.0)	974	971	982	1,013
Hesse	7.6	4.9 (- 2.7)	5.0 (- 2.6)	4.8 (- 2.8)	5.0 (- 2.5)	7.6 (0.0)	7.5 (0.0)	7.5 (0.0)	7.5 (0.0)	1,321	1,349	1,287	1,372
Mecklenburg-Western Pomerania	2.0	3.0 (1.1)	3.0 (1.1)	3.1 (1.1)	3.0 (1.1)	2.0 (0.0)	2.0 (0.0)	2.0 (0.0)	2.0 (0.0)	825	827	836	827
Lower Saxony	9.6	7.4 (- 2.2)	7.6 (- 2.0)	7.6 (- 2.0)	7.4 (- 2.2)	9.6 (0.0)	9.6 (0.0)	9.6 (0.0)	9.6 (0.0)	2,005	2,065	2,047	2,025
North Rhine-Westphalia	21.4	15.8 (- 5.6)	15.8 (- 5.7)	15.9 (- 5.5)	15.7 (- 5.7)	21.4 (0.0)	21.4 (0.0)	21.4 (- 0.1)	21.3 (- 0.1)	4,294	4,269	4,307	4,269
Rhineland-Palatinate	4.9	3.8 (- 1.2)	3.7 (- 1.2)	3.8 (- 1.2)	3.8 (- 1.1)	4.9 (0.0)	4.9 (0.0)	4.9 (0.0)	4.9 (0.0)	1,022	1,013	1,021	1,032
Saarland	1.2	3.3 (2.1)	3.2 (2.0)	3.3 (2.1)	3.3 (2.1)	1.2 (0.0)	1.2 (0.0)	1.2 (0.0)	1.2 (0.0)	896	875	893	902
Saxony	4.9	3.9 (- 1.0)	3.8 (- 1.0)	3.7 (- 1.2)	3.8 (- 1.1)	4.9 (0.0)	4.9 (0.0)	4.9 (0.0)	4.9 (0.0)	1,049	1,040	1,007	1,034
Saxony-Anhalt	2.6	3.2 (0.6)	3.1 (0.5)	3.1 (0.5)	3.1 (0.5)	2.6 (0.0)	2.6 (0.0)	2.6 (0.0)	2.6 (0.0)	876	837	852	844
Schleswig-Holstein^d^	3.5	9.9 (6.4)	9.8 (6.3)	9.8 (6.3)	9.9 (6.4)	3.5 (0.0)	3.5 (0.0)	3.5 (0.0)	3.5 (0.0)	2,685	2,665	2,665	2,697
Thuringia	2.5	3.1 (0.6)	3.1 (0.5)	3.1 (0.6)	3.0 (0.5)	2.5 (0.0)	2.5 (0.0)	2.6 (0.0)	2.5 (0.0)	849	832	842	826
**German citizenship** ^b^													
Yes	85.0	95.9 (10.9)	96.0 (11.0)	96.0 (11.1)	95.9 (10.9)	93.4 (8.4)	93.6 (8.7)	93.6 (8.6)	93.3 (8.3)	26,012	25,979	25,957	26,103
No	15.0	4.1 (- 10.9)	4.0 (- 11.0)	4.0 (- 11.1)	4.1 (- 10.9)	6.6 (- 8.4)	6.4 (- 8.7)	6.4 (- 8.6)	6.7 (- 8.3)	1,108	1,090	1,074	1,120
**Household size** ^c^													
Single-person household	25.4	21.4 (- 4.0)	21.4 (- 4.0)	21.3 (- 4.1)	21.6 (- 3.8)	25.7 (0.3)	25.7 (0.3)	25.7 (0.3)	25.7 (0.3)	5,794	5,783	5,762	5,867
Multi-person household	74.6	78.6 (4.0)	78.6 (4.0)	78.7 (4.1)	78.4 (3.8)	74.3 (- 0.3)	74.3 (- 0.3)	74.3 (- 0.3)	74.3 (- 0.3)	21,310	21,268	21,241	21,334

^a^Deviations from the reference value in percentage points

^b^Reference values according to population statistics for 2022

^c^Reference values according to the 2021 microcensus

^d^Additional samples were included for the federal states of Berlin and Schleswig-Holstein

Q = Questionnaire, CASMIN = Comparative Analysis of Social Mobility in Industrial Nation

**Annex Table 1:** Final AAPOR disposition codes, distribution and Response Rate 1 (RR1) in panel sub-wave 1 (Q2). Source: 2024 annual survey from the RKI Panel ‘Health in Germany’

Response rate 1 (RR1) sub-wave 1 (Q2) = 80.0 %	Frequency	Share (%)	Cumulative share (%)
**1.0 Returned questionnaire**			
1.1 Complete	37,558	79.95	79.95
1.2 Partial	654	1.39	81.34
**2.0 Eligible, non-interview**			
2.11 Refusal	84	0.18	81.52
2.112 Known-respondent Refusal (Hotline/E-Mail/Letter)	92	0.20	81.72
2.113 Empty questionnaire returned PAPI (Implicit Denial)^1^	6,122	13.03	94.75
2.2 Notification that respondent was unavailable during field period	2,382	5.07	99.82
2.3 Reminder letter: recipient deceased [returned mail. hotline]	58	0.12	99.94
2.31 Reminder letter: recipient deceased [returned mail. hotline]	20	0.04	99.98
2.32 Physically or mentally unable (information via hotline or general contact)	5	0.01	99.99
**3.0 Unknown eligibility, non-interview**			
3.10 Unknown (there has been no/never response from this address)	0	0.00	99.99
3.231 Invitation letter: Acceptance refused [returned mail -> postmark]	0	0.00	99.99
3.31 Invitation letter: Undeliverable/recipient cannot be determined at the specified address [returned mail]	0	0.00	99.99
**4.0 Not eligible, returned**			
4.1 Invitation letter (on first contact): Recipient moved [returned mail]	2	0.00	100.00
**Total**	**46,977**	**100.00**	**100.00**

^1^Code 2.113 was also assigned to CAWI participants who were known to have received the email in their inbox but did not start the online survey

Q = Quarter, AAPOR = American Association for Public Opinion Research. PAPI = Paper and Pencil Interview. CAWI = Computer Assisted Web Interview

**Annex Table 2:** Final AAPOR disposition codes, distribution and Response Rate 1 (RR1) in panel sub-wave 2 (Q3). Source: 2024 annual survey from the RKI Panel ‘Health in Germany’

Response rate 1 (RR1) sub-wave 2 (Q3) = 77.1 %	Frequency	Share (%)	Cumulative Share (%)
**1.0 Returned questionnaire**			
1.1 Complete	36,122	77.10	77.10
1.2 Partial^2^	12	0.03	77.13
**2.0 Eligible, non-interview**			
2.11 Refusal	115	0.25	77.38
2.112 Known-respondent Refusal (Hotline/E-Mail/Letter)	182	0.39	77.77
2.113 Empty questionnaire returned PAPI (Implicit Denial)^1^	7,698	16.43	94.20
2.2 Notification that respondent was unavailable during field period	2,681	5.72	99.92
2.3 Reminder letter: recipient deceased [returned mail. hotline]	3	0.01	99.93
2.31 Reminder letter: recipient deceased [returned mail. hotline]	30	0.06	99.99
2.32 Physically or mentally unable (information via hotline or general contact)	8	0.01	10 0.00
**3.0 Unknown eligibility, non-interview**			
3.10 Unknown (there has been no/never response from this address)	0	0.00	100.00
3.231 Invitation letter: Acceptance refused [returned mail -> postmark]	0	0.00	100.00
3.31 Invitation letter: Undeliverable/recipient cannot be determined at the specified address [returned mail]	0	0.00	100.00
4.0 Not eligible, returned			
4.1 Invitation letter (on first contact): Recipient moved [returned mail]	0	0.00	100.00
**Total**	**46,851**	**100.00**	**100.00**

^1^Code 2.113 was also assigned to CAWI participants who were known to have received the email in their inbox but did not start the online survey

^2^Due to a technical error in the online survey software, not all partially completed interviews could be retrieved

Q = Quarter, AAPOR = American Association for Public Opinion Research, PAPI = Paper and Pencil Interview, CAWI = Computer Assisted Web Interview

**Annex Table 3:** Final AAPOR disposition codes, distribution and Response Rate 1 (RR1) in panel sub-wave 3 (Q4). Source: 2024 annual survey from the RKI Panel ‘Health in Germany’

Response rate 1 (RR1) sub-wave 3 (Q4) = 74.6 %	Frequency	Share (%)	Cumulative Share (%)
**1.0 Returned questionnaire**			
1.1 Complete	35,483	74.65	74.65
1.2 Partial	303	0.64	75.29
**2.0 Eligible, non-interview**			
2.11 Refusal	11 9	0.25	75.54
2.112 Known-respondent Refusal (Hotline/E-Mail/Letter)	218	0.46	76.00
2.113 Empty questionnaire returned PAPI (Implicit Denial)^1^	8,878	18.68	94.68
2.2 Notification that respondent was unavailable during field period	2,423	5.10	99.78
2.3 Reminder letter: recipient deceased [returned mail, hotline]	60	0.12	99.90
2.31 Reminder letter: recipient deceased [returned mail, hotline]	44	0.09	99.99
2.32 Physically or mentally unable (information via hotline or general contact)	7	0.01	10 0.00
**3.0 Unknown eligibility, non-interview**			
3.10 Unknown (there has been no/never response from this address)	0	0.00	100.00
3.231 Invitation letter: Acceptance refused [returned mail -> postmark]	0	0.00	100.00
3.31 Invitation letter: Undeliverable/recipient cannot be determined at the specified address [returned mail]	0	0.00	100.00
**4.0 Not eligible, returned**			
4.1 Invitation letter (on first contact): Recipient moved [returned mail]	0	0.00	100.00
**Total**	**47,535**	**100.00**	**100.00**

^1^Code 2.113 was also assigned to CAWI participants who were known to have received the email in their inbox but did not start the online survey

Q = Quarter, AAPOR = American Association for Public Opinion Research, PAPI = Paper and Pencil Interview, CAWI = Computer Assisted Web Interview
